# Neurotoxic effects of the chemotherapeutic drug “methotrexate”: a literature review

**DOI:** 10.1097/MS9.0000000000002863

**Published:** 2024-12-23

**Authors:** Sarah Mshaymesh

**Affiliations:** a Department of Research and Education, Oli Health Magazine Organization, Kigali, Rwanda; b Faculty of Sciences, Haigazian University, Beirut, Lebanon

**Keywords:** cancer, chemotherapy, methotrexate, neurology, neurotoxicity, oncology, pharmacy, toxicity management, toxicity prevention, neuro-oncology

## Abstract

**Introduction:**

Methotrexate (MTX) is currently considered the go-to drug in chemotherapy, although it possesses neurological dangers. In this review paper, research and records of MTX-induced neurotoxicity in different types of adult and pediatric cancers will be discussed. The general description and function of MTX are still not completely known. How biological ratios in the body fluctuate with low and high methotrexate doses are still unknown as well. Therefore, this is a hot topic for experts, especially those active in neuro-oncology research. Scientists and physicians are now using different protocols to understand, control, manage, and prevent MTX-neurotoxic events.

**Aim:**

By evaluating various studies, case reports, and clinical trials, this comprehensive review seeks to clarify the mechanisms underlying MTX-induced neurotoxicity, highlight potential strategies for prevention and management, and guide future research in mitigating the adverse neurological effects of MTX treatment.

**Methods:**

Several databases were employed to gather this information, mainly PubMed.

**Results:**

The precise mechanisms of MTX-induced neurotoxicity remain unclear. Multiple hypotheses suggest involvement of genes related to neurogenesis, but no definitive cause has been identified. Regular patient monitoring, long-term follow-up, variable therapies, and early MRI detection are recommended.

**Conclusion:**

Despite advancements, no longitudinal studies have conclusively determined effective strategies to prevent MTX-induced neurotoxicity. Demographic factors and cancer types do not consistently predict neurotoxicity. Future research should focus on genetic counseling prior to chemotherapy, targeted monitoring protocols, and long-term patient follow-up to better understand and mitigate the neurotoxic impacts of MTX. Pharmacokinetics and advanced neuroimaging techniques are also essential for improving patient outcomes.

## Introduction

The World Health Organization considers methotrexate (MTX) as an essential medicine against many types of cancers^[1]^. This review explores MTX function, complications, symptoms, risks, management, and prevention in different cancer patients that range in age, gender, type of cancer, and treatment (i.e., dose, time, and combination of drugs), all of which result in varying levels of neurotoxicity. It is important to note that this review includes several case studies conducted at different time periods, however, it relies on specific locations and institutions due to their available resources and healthcare accessibility. The absence of longitudinal studies further highlights the need for ongoing research to establish a more comprehensive understanding of MTX’s neurotoxic effects and their implications for patient care.

### Neurotoxicity

Chemotherapy is a type of chemical treatment that helps destroy fast-growing cells, such as cancer cells. Like any drug or type of therapy, chemotherapy has its own side effects on its users. This happens because chemicals may also interfere with certain activities and mechanisms in the body. One of these side effects is cognitive dysfunction and neurotoxicity; sometimes referred to as “chemobrain”^[2,3]^. Neurotoxicity may potentially occur due to accumulation of nucleosides^[4]^, unmonitored administration of drugs, and/or high dose of certain chemotherapeutic drugs, such as Methotrexate^[1]^. Unfavorable neurological signs may lead to earlier treatment or dose adjustment^[5]^. High dose methotrexate (HDMTX)’s toxicity may lead to morbidity and occasional mortality, as it may also cause interruption in cancer treatment^[1]^. Some studies estimate the incidence of severe cognitive complications to be close to 100% in long-term survivor patients above the age of 60^[6]^. Therefore, managing MTX-induced neurotoxicity is essential to balancing cancer treatment efficacy and patient quality of life.

### Methotrexate

**Figure 1.**
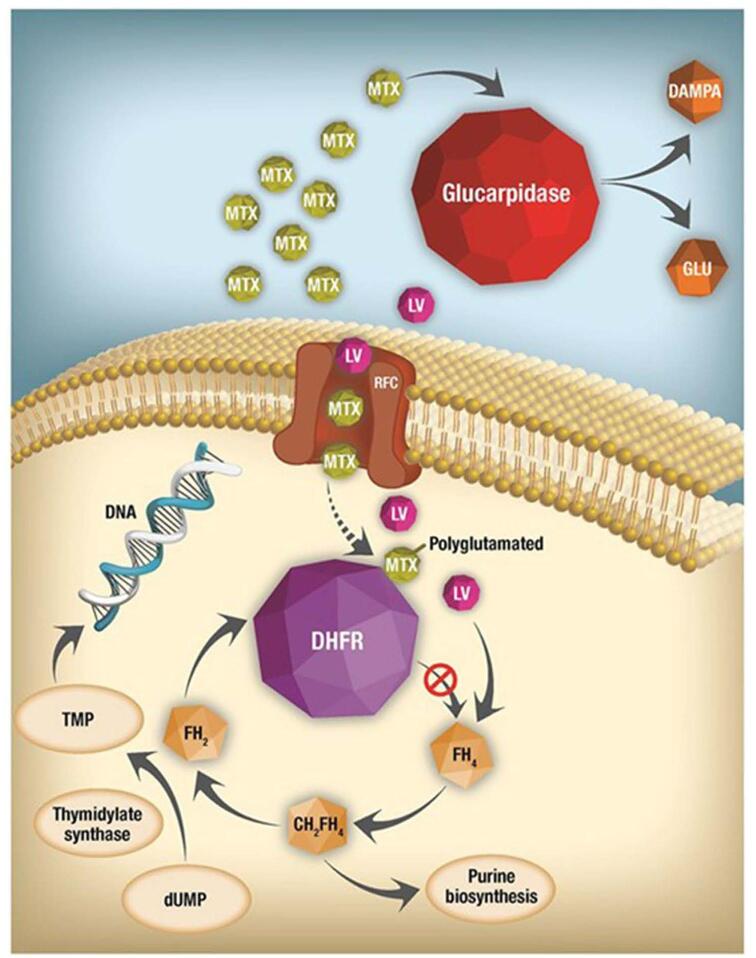
Methotrexate mechanism. Adapted from Howard et al. (2016). Preventing and Managing Toxicities of HDMTX^[1]^.

Methotrexate is an important cytostatic drug that is commonly used in chemotherapy for the treatment of osteosarcoma, acute lymphoblastic leukemia, non-Hodgkin lymphoma, histocytes, and especially in childhood cancers^[7]^. It is an antimetabolite that interferes with the metabolism of folic acid by having a binding affinity 1000-fold greater to dihydrofolate reductase^[1]^. MTX is polyglutamated as soon as it enters the cell, binds to dihydrofolate reductase, and inhibits the conversion of dihydrofolate to tetrahydrofolate, which is essential for the biosynthesis of purines and thymidine^[1]^. As illustrated in Figure 1, MTX enters the cell through reduced folate carrier (RFC). It is then polyglutamated and inhibits DHFR activity and prevents conversion of FH4 from FH2. DNA, RNA, and protein synthesis are inhibited by the lack of FH4. Leucovorin (LV) allows formation of FH4 in an attempt to rescue the cell. In other words, cells will be unable to divide and produce proteins due to MTX blocking tetrahydrofolate function. This results in elevated homocysteine and excitatory amino acid neurotransmitter metabolites^[7]^. HDMTX can cause hemiparesis, confusion, headache, emotional liability, slurred speech, choreoathetosis, and seizure^[5,8]^. While MTX is a vital treatment for various cancers, it can also lead to significant adverse effects.

## Types of cancers and MTX

Methotrexate is among many chemotherapeutic drugs that are used in both adult and pediatric cancers. What sets MTX apart is its potential to cause neurotoxic symptoms in patients, regardless of whether they had prior cognitive dysfunction. Proper administration of methotrexate, like any other chemotherapy, is crucial to avoid toxicity in any form. MTX doses range from 12 mg intrathecally and 20 mg/m^2^ orally, intramuscularly, or intravenously^[1]^. An intravenous dose of 500 mg/m^2^ or higher is considered HDMTX, which may be used in acute lymphoblastic leukemia (ALL), osteosarcoma, and lymphomas^[9]^. Higher doses may reach 33 000 mg/m^2^ intravenously as a weekly chemotherapeutic treatment in ALL^[10]^. “Leukoencephalopathy (LEP)” is the term used for MTX-associated neurotoxicity^[11]^. The following sections will discuss different types and cases of cancer (adult and child) and their relation to MTX neurotoxicity. It is important to note that knowledge of common toxicities helps differentiate between drug-induced CNS impairment and progression of fast-dividing cancer cells into the CNS^[6]^. If the knowledge and awareness are available, physicians are able to discontinue MTX as early as in 24 hours, such as in a case of a 21-year-old male (suffering from high-risk pre-B-cell ALL), who showed stroke-mimic symptoms. MRI demonstrated restricted diffusion involving subcortical white matter and splenium of the corpus callosum as a result of MTX and not the leukemia itself^[12]^. In summary, careful dosing and monitoring of this potent chemotherapeutic drug are crucial to preventing toxicity and securing safe treatment outcomes for both adult and pediatric patients.

### Acute toxic leukoencephalopathy

*The following data is mainly based on studies and case reports by authors Salkade, P and Lim, T*^[11]^.

Briefly, leukoencephalopathy is a structural modification of cerebral white matter in which great damage is inflicted on the myelin^[13]^. Therefore, “toxic leukoencephalopathy” is a result of exposure to toxins secondary to chemotherapy or cranial irradiation. Acute toxic leukoencephalopathy is considered in cases where a patient presents with recent onset of neurologic complications and exposure to a toxin (such as MTX) that may be harming the white matter.

**Figure 2.**
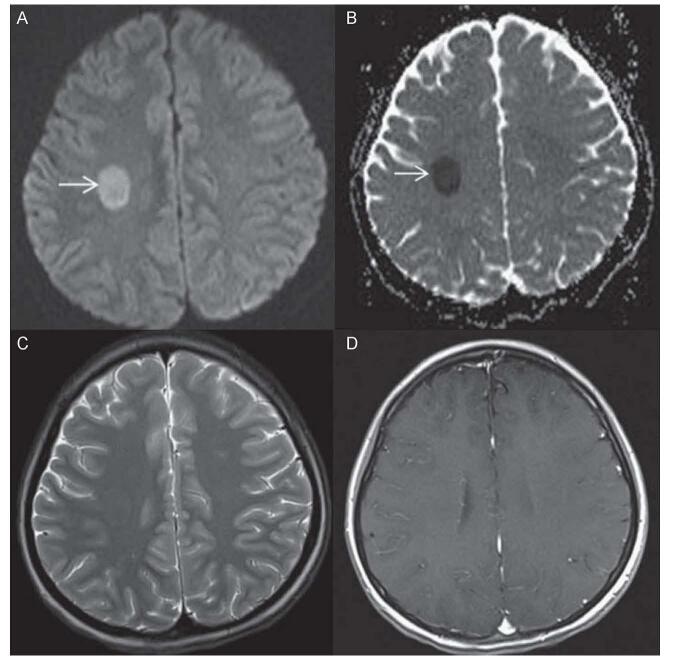
Diffusion-weighted imaging of MTX-LEP. (A) Focal restricted diffusion in right centrum semiovale. (B) Corresponding ADC map. (C) T2W1 displayed no abnormality in right centrum semiovale. (D) Post-gadolinium T1W1 displayed no pathological improvement in corresponding area. Modified from Salkade and Lim. (2012). MTX-induced acute toxic leukoencephalopathy^[11]^.

Acute lymphoblastic leukemia (ALL) is one of the most common childhood cancers. Physicians treat ALL patients with HDMTX, because it crosses the BBB. HDMTX can also be administered in an intrathecal manner to remove cancerous or leukemic cells from the CNS, which prevents CNS recurrence and hematologic relapses. Nevertheless, MTX is linked with neurotoxicity in these cases and affects the periventricular deep white matter area. MTX-induced ATL (also known as MTX-LEP) results in clinical manifestations that include acute neurological deficit, seizures, or encephalopathy – but the neurological deficits are temporary. Physicians use DW-MRI to identify acute strokes as well as differentiate acute stroke from non-stroke. Figure 2 depicts how DW-MRI detects the effects of MTX-induced leukoencephalopathy. In this case, a teenage Chinese female (diagnosed with ALL) is presented with left facial nerve paresis and 2 discrete episodes of left upper and lower limb weakness after receiving her second and third cycle treatment of HDMTX (IV and IT) and no cranial irradiation. DW-MRI was used after each episode of her neurological deficit and displayed focal restricted diffusion in right centrum semiovale. Within 3 days of symptom onset, the patient’s left sided focal neuro-deficit and facial nerve paresis almost fully subsided. After her neurological recovery, a follow-up DW-MRI showed that her right centrum semiovale’s restricted diffusion was almost completely resolved. The lesion, however, was not visible on concurrent T2W1^1^ and FLAIR sequences and did not show any contrast improvement on post gadolinium enhances T1W1^2^ sequences. Luckily, a 2-year clinical follow-up showed no residual neurological or intellectual issues.

Demyelination, myelin pallor, myelin vacuolation, axonal spheroids or macrophage infiltration, and necrosis are some of the histological findings of MTX-LEP. Although the pathophysiological mechanisms to MTX-LEP are still unknown, most scientists and physicians agree that it is multifactorial. One of the proposed understandings is that the altered choline-to-creatinine ratio is what causes MTX-related neurotoxicity in children due to the disturbances it causes in myelin metabolism or inhibition of glucose metabolism. The patient’s time of onset (of neuro-deficit) is recorded to be about 3 weeks from administration of HDMTX. It is important to note, though, that the incidence of MTX-LEP is from 3% to 10%, with variations in the dose, route of induction, and frequency of provided MTX^[14]^. Keeping in mind that HDMTX or high dose induction, IT route, young age, and association with cranial irradiation are some of the risk factors. As demonstrated in this case of MTX-induced toxic LE, early intervention with DW-MRI and vigilant monitoring leads to favorable outcomes, such as full recovery, even in the management of neurological deficits.

### Childhood acute lymphoblastic leukemia

*The following information is based on and retrieved from a research study by authors Bhowjani, D., Sabin, N., Pei, D. and others*^[15]^.

Authors assigned MTX as the reason behind any neurotoxic event if symptoms, such as seizure, bizarre behavior, stroke, or aphasia, occurred within 2 weeks of receiving said drug (IV or IT). Evaluations were made by a pediatric neurologist.

This study^3^ was performed on 408 children with ALL, 369 of which were exposed to five courses of HDMTX and 13 to 25 doses of triple intrathecal therapy (ITT). Table 1 provides key details on the study cohort. 278 of 369 patients received an upfront window treatment of HDMTX (1 g/m^2^, randomly assigned per 24 hours)^[16]^. Patients in low-risk arm were provided HDMTX (2.5 g/m^2^ per 24 hours) and patients in the high-risk arm were given 5 g/m^2^ per 24 hours, while maintaining plasma concentration adjustments^[17]^. 50 mg/m^2^ of leucovorin (LV) rescue were given at 44 hours, followed by 15 mg/m^2^ every 6 hours for 7 doses; 5 mg/m^2^ in low-arm and five doses beginning 42 hours of MTX administration in high-arm. Low-dose IV MTX (40 mg/m^2^) was administered post-second reinduction weekly, with dexamethasone/vincristine or cyclophosphamide/cytarabine monthly interruptions. In total, there were 68 to 116 MTX doses in continuation. GWAS was conducted for two different phenotypes: leukoencephalopathy (grade 0 *v* > 0) and clinical neurotoxicity (presence *v* absence).

**Table 1:** **Patient characteristic, neurotoxic events, and MTX rechallenge**.

**Patient No.**	**Age (years)**	**Sex**	CNS status*	**Therapy Arm†**	**MTX Before Event**	**Time Point in Therapy**	**Time from MTX to Events (days)**	**Neurotoxic Events**	**Duration of Event**	**Subsequent No. of High-Dose MTX Doses**	**Subsequent No. of ITTs**	**Prophylaxis**	**Recurrent Neurotoxicity**
1	13	M	CNS 2	Standard	High-dose MTX, ITT	Consolidation course one	4	Seizure (tonic clonic)	2 minutes	3	20	No	
2	3	M	CNS 1	Low	Low-dose MTX, ITT	Continuation week 40	9	Seizure (complex partial)	24 hours	0	0	NA	
3	5	M	CNS 1	Standard	High-dose MTX, ITT	Consolidation course two	3	Seizure (tonic clonic)	5 minutes	2	13	Leucovorin after ITT	
4	15	M	CNS 1	Standard	High-dose MTX, ITT	Consolidation course two	10	Stroke-like	72 hours	0 (low dose)	7	No	
5	4	M	CNS 1	Standard	ITT	Continuation week 12	7	Ataxia	4 weeks	0	8	Leucovorin after ITT	
6	10	F	CNS 1	Standard	High-dose MTX, ITT	Consolidation course three	8	Seizure (complex partial)	24 hours	1	11	Leucovorin after ITT	
7	11	M	CNS 2	Standard	High-dose MTX, ITT	Consolidation course one	11	Seizure (tonic clonic)	20 minutes	3	13	Leucovorin after ITT	
8	2	F	CNS 2	Low	Low-dose MTX, ITT	Continuation week 36	8	Seizure (complex partial)	24 hours	0	3	No	
9	16	M	CNS 1	Standard	ITT	Continuation week 13	9	Stroke-like	24 hours	0	8	Leucovorin after ITT	
10	14	F	CNS 1	Standard	High-dose MTX, ITT	Consolidation course one	7	Stroke-like	5 hours	1 (omit 2)‡	9	Aminophylline	Headache, confusion
11	5	M	CNS 1	Standard	ITT	Continuation week 29	7	Seizure (complex partial)	7 days	0	11	No	
12	12	M	CNS 1	Standard	High-dose MTX, ITT	Consolidation course one	10	Stroke-like	48 hours	3‡	11	Aminophylline	
13	18	M	CNS 1	Standard	High-dose MTX, ITT	Consolidation course one	10	Stroke-like (and seizure)	8 hours	3	20	No	Stroke (CNS thrombus)
14	17	F	CNS 1	Standard	Low-dose MTX, ITT	Continuation week 88	11	Stroke-like	36 hours	0	1	No	

* CNS1, <5 WBC/μL of CSF without blasts; CNS2, <5 WBC/μL of CSF with any blasts. ^†^ Details on risk stratification described by Pui et al^[18]^. ^‡^ Second high-dose MTX and ITT given 1-2 weeks apart. Adapted from Bhojwani et al. (2014). Methotrexate-Induced Neurotoxicity and Leukoencephalopathy in Childhood Acute Lymphoblastic Leukemia^[15]^.

**Figure 3.**
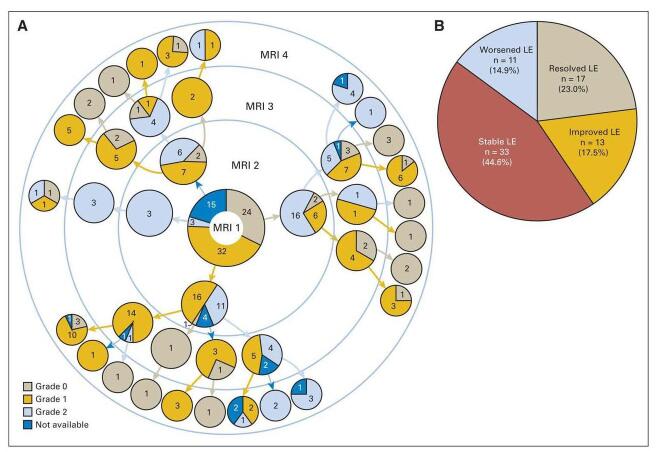
MRI for 74 patients with LE. (A) Grading of MRI for patients at four screening time points. Patient number and LE grades are indicated in pie charts. (B) MRI changes of LE over time; resolved, improved, worsened, or stable with their respective percentages. Adapted from Bhojwani et al. (2014). Methotrexate-Induced Neurotoxicity and Leukoencephalopathy in Childhood Acute Lymphoblastic Leukemia^[15]^.

The major difference in this ALL study as compared to other ones is that 13 patients were rechallenged with HDMTX or ITT, except for Patient No. 2 as he only required one more dose of ITT. Results showed that 14 patients developed MTX-associated neurotoxicity. Of 13 patients rechallenged with IT HDMTX, 12 did not experience neurotoxic recurrence. Figure 3 outlines MRI findings at four screening time points for 74 patients with LE. Leukoencephalopathy was found in 73 of 355 asymptomatic children (persisted in 74%) and in all symptomatic children (persisted 58%). Additionally, a high 42-hour MTX-LV rescue ratio was linked with an elevated risk of leukoencephalopathy. GWAS demonstrated polymorphisms in neurodevelopmental-rich genes (*GSTP1*^[19]^, *MTHFR*, and *SHMT1*^[20]^) with high-risk mechanisms of neurotoxicity. These results conclude that MTX-neurotoxicity is temporary and that most patients are eligible to receive subsequent MTX without having to worry about recurrences of acute or subacute effects. Polymorphisms in neurogenesis-related genes may be associated with MTX-neurotoxicity. 1/5 asymptomatic patients and all symptomatic children develop leukoencephalopathy, which can carry on until therapy ends. The only limitation in this research was the absence of a true baseline MRI. Overall, although most patients can safely continue treatment, genetic factors and delay of leucovorin rescue could elevate the risk of leukoencephalopathy.

### Primary CNS lymphoma

*The general description and overview on PCNSL are based on a research article by authors Grommes, C. and DeAngelis, L.* ^[21]^

Primary central nervous system lymphoma (PCNSL) is a rare form of non-Hodgkin’s lymphoma that is restricted to the brain, eyes, and CSF. This type of tumor favors both chemotherapy and radiation therapy, but survival levels are usually low as compared to tumors outside the CNS. Although the addition of methotrexate to PCNSL treatment showed improvement during the past decades, recent studies have associated it with late neurotoxicity. The limited number of randomized trials for PCNSL has resulted in numerous unresolved questions. Immunocompetent patients (4%-6%) are most likely to develop PCNSL. They tend to develop neurologic symptoms within weeks, including focal neurologic deficits (56%-70%), mental status and behavioral changes (32%-43%), symptoms of increased intracranial pressure (headaches, nausea, vomiting, papilledema, 32%-33%), and seizures (11%-14%)^[22]^.

**Figure 4.**
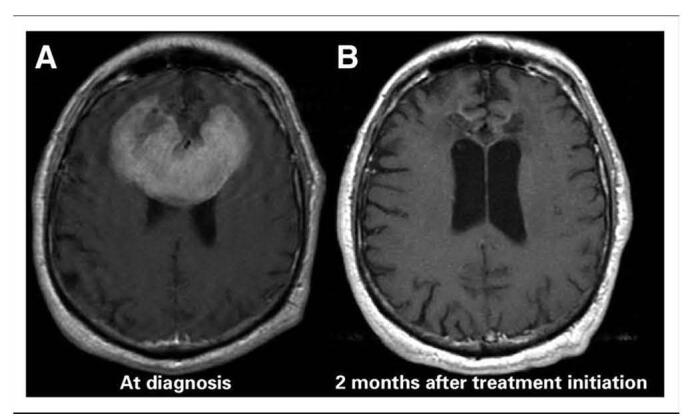
Brain MRI PCNSL. (A) MRI (T1+ gandolinium) displays large, frontal-enhancing brain lesion. (B) Follow-up MRI displays resolution of the large lesion after 2-month treatment with HDMTX. Adapted from Grommes and DeAngelis. (2017). Primary CNS Lymphoma^[21]^.

To detect the extent of PCNSL and what may be causing neurological toxicities, it is best to establish a baseline. Experts agree that the best treatment for PCNSL is HDMTX^[23]^ and in fact refer to it as the “backbone of multimodal therapy” in addition to other chemotherapeutic drugs with or without radiation. This is demonstrated in Figure 4, which shows an MRI imaging of a frontal brain lesion 2 months post-HDMTX therapy. They believe so because HDMTX administration is able to penetrate the BBB. However, some authors doubt the efficacy of radiotherapy in prolonging survival in PCNSL patients^[24]^. New research proposes that the combination of HDMTX and LV prevents bone marrow and systemic organ damage. Furthermore, rechallenge of HDMTX in recurrent PCNSL seems to be successful as it led to an overall response rate of 85% to 91% with median overall survival of 41 to 62 months^[21]^. It especially displayed success when there was a long period of remission. A case report on PCNSL patients (with a baseline comatose neurological state) concluded that a poor neurocognitive condition before treatment is a poor indicator for the post-treatment neuro-outcome across demographical factors^[23]^. The study was done on 3 patients of different age and race. Despite the success of HDMTX in treating PCNSL, unresolved questions about the efficacy of radiotherapy and the potential neurotoxicity highlight the need for further investigation.

#### MTX-induced neurotoxicity

*The following information is based on a case report by authors Oarbeascoa, G., Rodriguez-Macias, G., and others.* ^[25]^

A 21-year-old male diagnosed with stage 4 large B-cell lymphoma presented a CNS relapse. He endured sudden left-side hemiparesis while receiving IV MTX using an Ommaya reservoir and systemic MTX. According to the authors, “intraventricular administration of methotrexate (MTX) using an Ommaya reservoir^4^ is a useful therapeutic maneuver for malignant CNS involvement in patients with hematological malignancies.” FLAIR and MRI^5^ findings were positive among all other negative diagnostic tests and demonstrated hyperintensity in the basal ganglia and restricted diffusion within the corresponding area that followed the Ommaya catheter. Hence, HDMTX administration resolved the syndrome.

**Figure 5.**
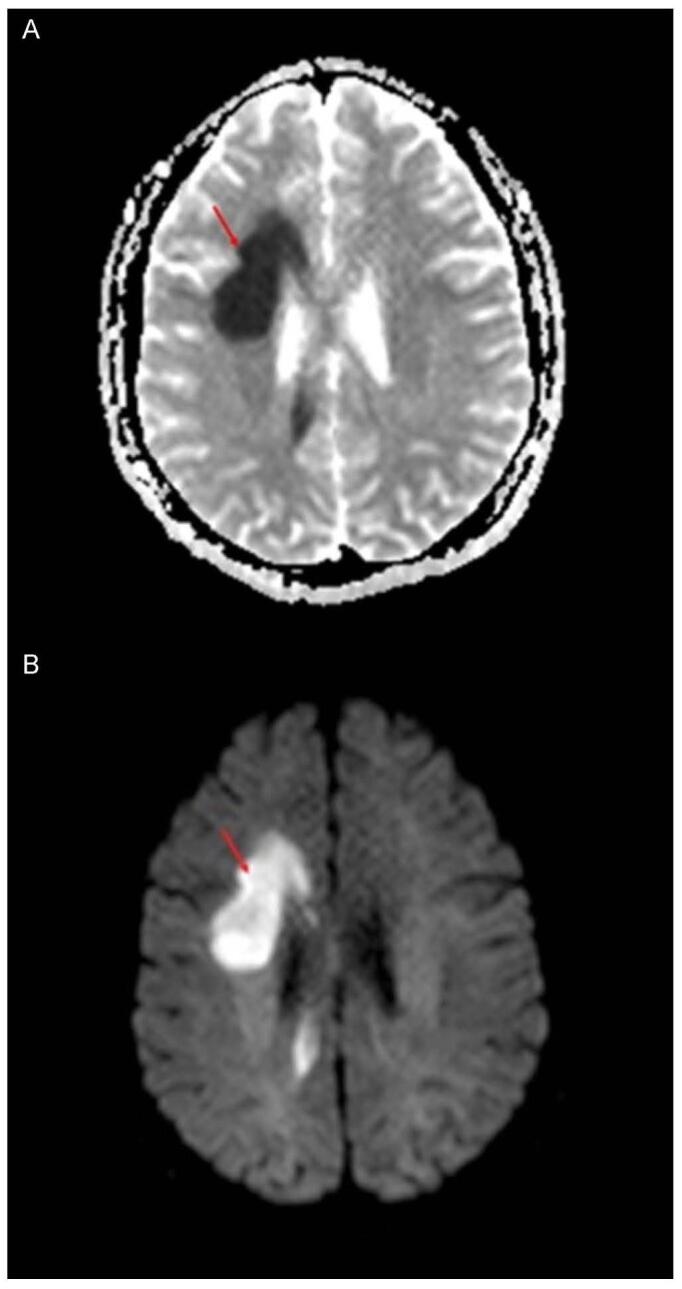
Brain MRI in corona radiata. (A) Diffusion ADC displaying intense restricted diffusion. (B) DWI displaying intense restricted diffusion. Modified from Oarbeascoa et al. (2019). MTX-Induced Subacute Neurotoxicity Surrounding an Ommaya Reservoir in a Patient with Lymphoma^[25]^.

MTX-associated neurotoxicity is an adverse reaction to systemic and IT induction of the drug. In the past, neurotoxicity would be linked to an incorrectly placed or malfunctioning Ommaya catheter. Recently and in the presented case, it is suggested that the catheter track was more susceptible to methotrexate toxicity. MRIs were constantly performed to monitor the 21-year-old and he later continued his IV MTX and systemic chemotherapy without recurrence of symptoms. A control MRI was done 15 days after resolution and indicated normalization of the restricted diffusion in the ADC map, in addition to more white substance lesions and hyperintensity in FLAIR/T2 and hypointensity in T1. Figure 5 shows the brain MRI images of the restricted diffusion in the *corona radiata*. In the end, the patient was scheduled for a stem cell transplantation with post-transplant treatment.^6^

For patients receiving MTX, acute or subacute neurotoxicity may be rare. However, MRI scans with highly restricted diffusion are crucial for those displaying neurological disturbances during MTX administration. All factors must be taken into consideration, even when IV catheters are placed correctly and functioning properly, because they may present as pre-disposing factors. Fortunately, proper management, MRI monitoring, and awareness of risks, such as catheter track susceptibility to MTX toxicity, are crucial for ensuring the safe continuation of MTX induction.

#### Chemotherapy with OBBBD

A new study of its kind was made at Centre hospitalier universite de Sherbrooke (November 1999–May 2018) regarding the management of PCNSL using MTX, but with an osmotic blood-brain barrier disruption (achieved using a 25% mannitol intra-arterial infusion)^[26]^. Current treatments typically use IV delivery route for MTX with no consideration for the BBB, which prevents most medication from entering the CNS and, consequently, challenges CNS pathology treatment^[27-30]^. The authors then came up with this new approach using CIAC coupled with osmotic blood–brain barrier disruption (OBBBD) using two different HDMTX protocols. The first protocol (1999–2007) was using HDMTX 5 g IA with etoposide phosphate (400 mg/m^2^ IV) and cyclophosphamide (660 mg/m^2^ IV), while the second (2008–2018) was HDMTX 5 g IA with carboplatin (400 mg/m^2^ IA). The patients were followed clinically (complete blood and platelet count and electrolytes) and radiologically (before each cycle) monthly until remission or death. Neurological and physical examination were assessed pre-cycle.

Unless any abnormal activity was revealed on imaging, patients were treated every 4 weeks (1 cycle) for 12 cycles, alternating between vascular distributions from one cycle to another. Depending on the tumor’s location, catheterization in internal carotid artery (either the right or left) or dominant vertebral artery was done using a transfemoral approach, and later obtaining a diagnostic angiogram. The OBBBD was executed in the selected vascular distribution for 30 seconds, filling the vascular distribution to maximize mannitol-endothelial cells reach. Rate of infusion ranged from 3 to 6 mL/s and IA-infused chemotherapy. In a total of 44 patients, 15 were exposed to regimen 1 and 29 to regimen 2.

Complete response was achieved in 34 patients (79%) at a 7.3 months median. Adverse reactions included neutropenia (20%), seizures (11%), and stroke (2%). Based on neurocognitive assessments, authors were able to hypothesize that repeated OBBBD treatments with IA HDMTX are unlikely to result in significant neurotoxicity compared to other HDMTX regimens and studies. Table 2 summarizes the adverse effects of OBBBD and HDMTX-neurotoxicity. Thus, these findings suggest that integrating OBBBD with IA HDMTX is an effective strategy for managing PCNSL, achieving high response rates with minimal neuro-related risks.

**Table 2:** **Adverse effect of OBBBD and HDMTX-neurotoxicity**.

Seizures	5 (11%)
Grade 1 (focal seizure)	0
Grade 2 (generailzed seizure)	4 (9%)
Grade 3 (multiple, medication-resistant seizures)	0
Grade 4 (life-threatening seizure)	1 (2%)
Grade 5 (death)	0

Modified from Iorio-Morin et al. (2021). Management of PCNSL Using Intra-Arterial Chemotherapy with OBBBD: Retrospective Analysis of the Sherbrooke Cohort^[26]^.

### Case report: osteosarcoma

*The following data on this osteosarcoma case is retrieved from a case report by authors Daniel Almeida do Valle et al*^[7]^.

One of the treatments for osteosarcoma is high dose methotrexate (HDMTX)^[20]^, which may cause neurological side effects that range from acute, subacute, and chronic complications^[5]^. Therefore, physicians may provide what is known as MTX-rescue, Leucovorin (LV). A rare form of MTX neurotoxicity associated with osteosarcoma is stroke-like encephalopathy. It displays sudden onset of focal neurological complications^[8]^ that occur quickly within days to weeks after MTX administration. A recent study suggests that 3.8% of all MTX-administered patients developed a related subacute neurotoxic event, which were discovered by MRI in 20.6% of asymptomatic individuals and in all symptomatic patients^[15]^.

This specific case is about a 15-year-old male patient diagnosed with osteosarcoma (of the right distal tibia and pulmonary metastasis). His neoadjuvant chemotherapy included cisplatin (60 mg/m^2^ per day for 2 days) and doxorubicin (37.5 mg/m^2^ per day for 2 days), while alternating with HDMTX (12 g/m^2^) in a 6-week cycle. After 24 hours, LV (15 mg) was given each 6 hours after every cycle of HDMTX. The patient kept receiving this treatment until safe MTX plasma concentrations were observed. Of course, MTX administration and plasma levels were carefully monitored. After the fourth cycle, MTX plasma levels showed concentrations of 6.26 μmole/L (24 hours), 0.78 μmole/L (48 hours), and 0.13 μmole/L (72 hours). After 12 days of treatment, the teenager presented with odd behavior, violence, and psychomotor distress, but was aware of time and space. An urgent CT scan was performed due to sudden onset of left-sided upper and lower limb paresthesia with ipsilateral hyporeflexia, with no face involvement. Nonetheless, there were no identified abnormalities through imaging, physical examination, and lab exams (hematology, viral serology, blood chemistry, and CSF). The patient began complaining of right-sided hemiparesis, but with stable vital signs and mental status.

**Figure 6.**
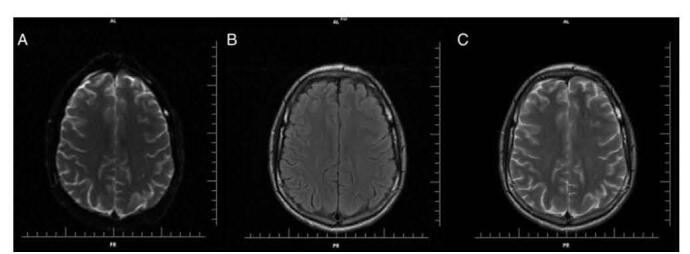
Brain MRI osteosarcoma (30 days after onset). (A) DWI. (B) Axial T2/Flair. (C) Axial T2. Adapted from Valle et al. (2016). Stroke-like encephalopathy following high-dose IV MTX in an adolescent with osteosarcoma: a case report^[7]^.

Physicians then performed a gadolinium-enhanced MRI, which displayed symmetrical hyperintense DWI signals and decreased ADC in the parietal lobe white matter, but more prominent on the left side. Nonetheless, the cortical area and deep gray matter were not affected and no change in signal were shown on FLAIR and T2 images. Finally, MTX-induced stroke-like encephalopathy was implied due to the absence of vascular abnormalities, which propose transient cytotoxic edema of the white matter. 30 days after presentation of the first abnormality, MRI showed complete resolution and, again, no signal was seen on T2 or FLAIR. Figure 6 shows the MRI findings. The patient continued to be neurologically asymptomatic.

Unfortunately, the pathophysiology mechanism is not yet understood properly, but it is suggested to be multifactorial. Scientists and physicians propose several possibilities for such MTX neurotoxicity that include chronic folate reduction in the brain, increased excitatory amino acids, elevation in homocysteine affecting on n-methyl-D aspartate (NMDA) in biopterin metabolism^[31]^, and adenosine metabolism also due to excess homocysteine. Increased risk of leukoencephalopathy is associated with HDMTX levels (within 2 days) and higher homocysteine concentrations^[15]^. Additionally, single nucleotide polymorphisms have been shown to influence symptomatic neurotoxicity and leukoencephalopathy risk. This case reinforces the importance of diligent monitoring and imaging, as well as the need for further research to advance the prevention and treatment of rare MTX-induced complications, such as stroke-like encephalopathy.

## Cognitive impairment in rodents

*The following research study is based on the work of authors Berlin, C., Lange, K., Lekaye, H., Hopland, K., Phillips, S., Piao, J., and Tabar, V.* ^[3]^

Because the methotrexate mechanism that is causing neurotoxicity is still unclear, a research was done on rodents instead to study the manifestations of MTX in the brain (both white and grey matter, because the focus was on grey matter in the past). The results from this study suggest that 6- and 16-months post-chemotherapy, rats that were treated with MTX showed a “significant and permanent” decrease in oligodendrocyte number and their progenitors in the white matter, corpus callosum volumes, and myelin basic protein. The authors were able to deduce these results through the rats’ performance on tasks associated with MWM and NOR. The rats also displayed a temporary decrement in white matter microglia (at 3 months) and hippocampal neural progenitors (at 3 and 6 months). Imaging indicated remarkably decreased fractional anisotropy values in the callosum body, genu, splenium, and fimbria (which is among areas that have not been previously assessed). In summary, all the collected results demonstrate a permanent negative effect of MTX (specifically HDMTX) on the oligodendrocyte populations and white matter, leading to cognitive dysfunction due to impaired ability in myelin turnover. This specifically is one of the concerns that cancer patients (or survivors) have to deal with post-chemotherapy; the development of neurological impairment related to chemotherapeutic drugs, such as methotrexate. In fact, it is so common that scientists and physicians now term these effects of chemotherapy-induced cognitive impairment (CICI) as “chemobrain”^[32]^. Mechanisms of CICI are currently under investigation and this too has brought the attention on investigating the mechanism of MTX neurological effects, especially that HDMTX is the modern treatment for ALL, non-Hodgkin’s lymphoma, pediatric osteosarcomas, and primary CNS lymphoma^[33]^. In addition to treatment, it is also currently used for the management of leptomeningeal metastases (administered IT)^[34]^.

In this study, the authors used Sprague-Dawley rats, male and female, aged 3 weeks and randomly assigned them into two groups (Control n = 18; MTX rats n = 17). MTX rats were administered a dose of 200 mg/kg/week for 4 weeks via intraperitoneal injection with a final dose of 800 mg/kg. Additionally, leucovorin rescue was administered as well to block MTX-induced acute neurotoxicity. During the treatment phase, no adverse effects from methotrexate were recorded. The protocol the authors used was the following: two tests (MWM and NOR), stain for Ki67, Olig2, Iba1, and MBP, stereology was performed using Stereo Investigator 2017, and diffusion tensor imaging to measure regional FA, AD, RD, and MD.

**Figure 7.**
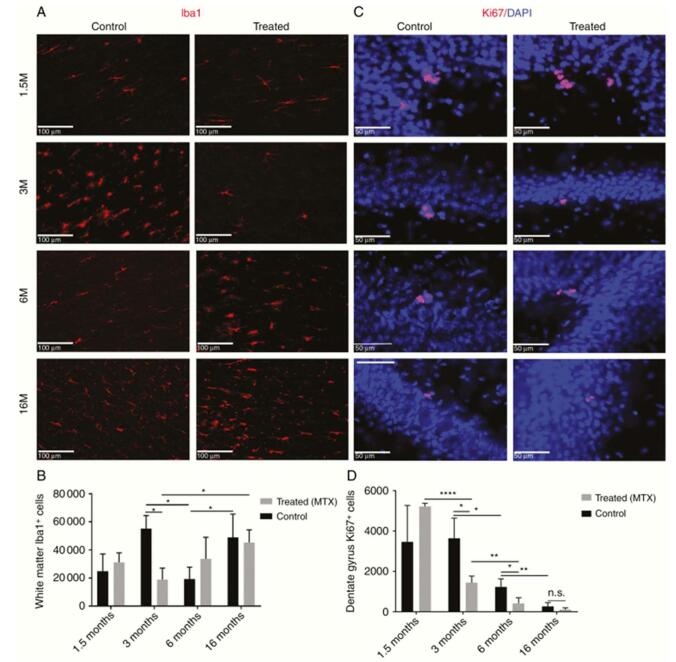
MTX effects on microglia and neurogenesis in rodents. Adapted from Berlin et al. (2020). Long-term clinically relevant rodent model of methotrexate-induced cognitive impairment^[3]^.

The authors were able to deduce, in addition to the information written above, that systemic doses of MTX resulted in a significant decrease in the extent of myelination and associated corpus callosum volume. Decrease in FA demonstrated the microstructural destruction to the white matter in certain areas. The behavioral tests revealed the hippocampal neurogenesis and long-term memory complications [MWM] and the deficits in the frontal-lobe related process (i.e., attention) and short-term memory [NOR]. These are common symptoms of CICI, but the significant rate of proliferation in the dentate gyrus was established to be a result of MTX effects on the brain. The behavioral differences between the two groups of rodents remained later in their life and even multiple months after the last methotrexate dose. DTI test suggested that impact on the white matter, including fimbria.^7^ Consequently, damage to the hippocampal pathways contributed to memory impairment of spatial orientation and, later, dysfunction of multitasking. The anti-inflammatory effect by methotrexate caused suppression in microglia population. However, alterations in microglia were recovered later and achieved a peak (at 16 months). In other words, microglia numbers were similar to those of normal rats. Figure 7 illustrates the effects of MTX on microglia and neurogenesis in rodents. The reason why microglia were not affected is because MTX also functions as an immunomodulator. Overall, this study reveals the long-term adverse effects of MTX on cognitive functions, oligodendrocytes, and white matter at different time intervals.

## Management and prevention

Management and prevention protocols vary upon tumor or cancer type. When a certain toxicity occurs in the body, usually it is the excretory system that aids in getting rid of these toxic wastes. Therefore, a malfunction in the kidneys, for example, may result in these toxic wastes accumulating in the body and causing further damage. Any form of methotrexate toxicity in the body must be excreted similarly. However, when there is a delay in MTX excretion, these elevated harmful concentration levels can lead to significant morbidity and mortality^[10]^. It is important to assess renal function before, during, and after administration of HDMTX^[1]^. Any abnormal values (elevation or decrease) in serum creatinine concentration and other parameters suggest potential renal dysfunction, which in turn explains that there is a delay in MTX elimination and a higher risk of toxicity (i.e., neurotoxicity). Increasing the rate of intravenous fluids, using an alternative drug that does not interfere with MTX clearance, or even stopping methotrexate (in extreme cases) are all different approaches that a physician may use if they identify early warnings^[1]^.

Protocols may vary when measuring serum MTX. One protocol suggests that after starting MTX infusion, plasma MTX concentrations may be appropriate at 24, 48, and 72 hours. Another require serum MTX to be measured at 36 hours (12 hours at the end of a 24-hour infusion) or at 42 hours (from the start)^[35]^. Consequently, leucovorin doses are adjusted accordingly. *(See* Table 3 *for an overview of MTX and HDMTX protocols in chemotherapy).* Serum MTX concentrations should be monitored with constant modifications in hydration, alkalinization, and leucovorin rescue until concentration level reaches >0.05–0.1 μM^[10]^. Nonetheless, urine pH and output, serum creatinine, and twice-daily examination of mucosal membranes should be monitored for areas where MTX levels cannot be^[1]^. Accordingly, the physician may then proceed to provide the patient with HDMTX, if applicable.

**Table 3:** MTX and HDMTX protocols in chemotherapy.

Study, year* [reference]	Methotrexate dose	Duration of methotrexate infusion (hours)	Leucovorin rescue dose	Time from start of methotrexate infusion to first leucovorin dose (hours)
Acute lymphoblastic leukemia				
Takeuchi et al., 2002 [98]	100-mg/m^2^ bolus, then 500 mg/m^2^ per hour	4	15 mg every 6 hours × 8 doses	28
Linker et al., 2002 [99]	220-mg/m^2^ bolus, then 60 mg/m^2^ per hour × 36 hours	36	50 mg every 6 hours	36
Hill et al., 2004 [100]	6 g/m^2^ (age < 4 yr)	10% bolus, remainder over 23 hours	15 mg/m^2^ every 3 hours, then every 6 hours when serum methotrexate <2 × 10^6^ *µ*M	36
	8 g/m^2^ (age > 4 yr)			
Pui et al., 2007 [58]	2 g/m^2^	2	10 mg/m^2^ every 6 hours	44
Zhang et al., 2014 [101]	3–5 g/m^2^	24	15 mg/m^2^ every 6 hours, then pharmacokinetically guided to serum methotrexate 0.1 *µ*mol/L	36
Osteosarcoma				
Souhami et al., 1997 [102]	8 g/m^2^ (age ≥ 12 yr)	Not specified	12 mg/m^2^ i.v. or 15 mg/m^2^ p.o. every 6 hours for 10 doses	24
	12 g/m^2^ (age < 12 y)			
Fuchs et al., 1998 [103]	12 g/m^2^ (maximum, 20 g)	Not specified	15 mg/m^2^ every 6 hours for 12 doses	Not specified
Bacci et al., 2001 [104]	12 g/m^2^ (escalated to 14 g/m^2^ if the 6-hour serum methotrexate was <1 *µ*mol/L)	6	15 mg every 6 hours for 11 doses	24
Goorin et al., 2003 [105]	12 g/m^2^	4	15 mg every 6 hours for 10 doses	24
Ferrari et al., 2005 [106]	12 g/m^2^	4	8 mg/m^2^ every 6 hours for 11 doses	24
Non-Hodgkin lymphoma				
Koller et al., 1997 [107]	200 mg/m^2^ over 30 min, then 800 mg/m^2^	24	50 mg i.v. for one session, then 15 mg p.o. every 6 hours or as methotrexate concentrations define	36
Khouri et al., 1998 [108]	200 mg/m^2^ over 30 min, then 800 mg/m^2^	24	50 mg i.v. for one session, then 15 mg p.o. every 6 hours or as methotrexate concentrations define	36
Thomas et al., 2004 [109]	200 mg/m^2^ over 30 min, then 800 mg/m^2^	24	50 mg i.v. for one session, then 15 mg p.o. every 6 hours or as methotrexate concentrations define	36
Primary central nervous system lymphoma				
Batchelor et al., 2003 [110]	8 g/m^2^	4	Pharmacokinetically guided until serum methotrexate <1 × 10^6^ *µ*M	24
Wright et al., 2015 [46]	2,000/5,000 mg/m^2^	24	15 mg/m^2^ every 6 hours for a total of 5 doses	24
Dalia et al., 2015 [111]	8 g/m^2^	Not specified	Not specified	Not specified

Adapted from Howard et al. (2016). Preventing and Managing Toxicities of HDMTX**^[1]^.** *The references in the table are based on the reference list from Howard et al. ^[1]^.

Authors offer multiple suggestions for management of neurotoxicity (i.e., imagining, monitoring, genetic counseling, etc.). Strict monitoring and prompt intervention are highly important for the stimulation of MTX excretion and prevention of toxicity^[1]^. They also help physicians assess if the patient is able to be administered HDMTX or not^[1]^, because CNS toxicity may occur after HDMTX. If a patient develops CNS toxicity, all potential neurotoxins should be discontinued^[5]^, and an MRI should be performed^[1]^. Increased MTX to LV ratios (42 hours) suggest higher risk for CNS toxicity, as well as polymorphisms. Genetic proposals concerning an increased risk of cognitive dysfunction, CNS toxicity, and neurotoxicity are said to be due to polymorphisms in neurodevelopment-associated genes, such as *TRIO, PRKG1, ANK1, COL4A2, NTN1, ASTN2, GSTP1, MTHFR*, and *SHMT1*^[15]^. Another genetic suggestion states that complications with HDMTX clearance and toxicity are associated with a polymorphism of the gene SLCO1B1, which encodes for a hepatic solute carrier organic transporter that interferes with MTX^[15,36,37]^. As a result, genetic counseling (prior to chemotherapy), early intervention, thorough imaging, proper dosing, and continuous monitoring contribute to patient safety and minimize overall toxicity.

## Limitations

The understanding of MTX’s neurotoxic effects is subject to several limitations. Key challenges include the absence of large randomized clinical trials, inadequate long-term follow-up, and a limited range of pharmacologic therapies. Additionally, there are insufficient studies specifically assessing neurotoxicity across diverse patient populations, particularly among those who may experience cognitive impairment years after MTX treatment. Concurrent treatments and variability in dosing regimens further complicate the establishment of clear causative relationships. Many existing studies are conducted in countries and institutions with ample resources, which may limit the generalizability of the findings to diverse populations with varying access to healthcare. Factors such as patient age, medical history, genetic predispositions, and prior cognitive function are not consistently considered, making it difficult to isolate MTX’s specific impact on neurotoxicity. These challenges highlight the necessity for more comprehensive longitudinal studies to clarify the extent and mechanisms of MTX-induced neurotoxicity.

## Conclusion

The different MTX protocols and approaches to cancer therapy signify that experts are trying to find a way to treat their patients with the least possible risks, whether they are neurocognitive-related or not. It is still unknown what is precisely causing an aggressive or, in other cases, slight neurotoxicity within the methotrexate mechanism in the brain. There are several hypotheses, but many revolve around the same concepts, such as genes related to neurogenesis. Physicians and scientists agree that there are possible measures that may restrict, manage, or prevent MTX-induced neurotoxicity like, proper monitoring of MTX-administered patients and performing MRIs for early detections. New research and advanced medicine have given experts the chance to perform new studies. They are now opting for completely new approaches using advanced technology to infer past mistakes, new protocols, genetic interrelation, and passing beyond barriers within the brain. Physicians and scientists are now trying to combine forces of pharmacokinetics, genetics, immunology, oncology, neurology, and neuroimaging to have a better understanding of methotrexate mechanism and associated neurotoxic symptoms.

## Data Availability

Not applicable.

## Footnotes

^1^
Image in Figure 2.

^2^
Image in Figure 2.

^3^
Study took place in St June Children’s Research Hospital from June 2000 to October 2007.

^4^
Catheter inserted within brain.

^5^
The main diagnostic tool in MTX-induced toxicity.

^6^
Myeloablative cyclophosphamide as prophylaxis for graft-versus-host disease (GvHD).

^7^
Fimbria contain afferent and efferent hippocampus pathways.
